# Cannabinoids in Neurology - Position paper from Scientific Departments from Brazilian Academy of Neurology

**DOI:** 10.1590/0004-282X-ANP-2020-0432

**Published:** 2021-05-24

**Authors:** Sonia Maria Dozzi BRUCKI, Tarso ADONI, Carlos Mauricio Oliveira ALMEIDA, Daniel Ciampi de ANDRADE, Renato ANGHINAH, Luciana Mendonça BARBOSA, Rodrigo BAZAN, Alzira Alves de Siqueira CARVALHO, William CARVALHO, Paulo Pereira CHRISTO, Marcus Della COLETTA, Adriana Bastos CONFORTO, Ylmar CORREA-NETO, Eliasz ENGELHARDT, Marcondes Cavalcante FRANÇA, Clelia FRANCO, Felipe VON GLEHN, Helio Rodrigues GOMES, Caroline Gomes de Barros HOULY, Alexandre Ottoni KAUP, Fernando KOWACS, Aline KANASHIRO, Victor Gonçalves LOPES, Débora MAIA, Maria MANREZA, Alberto Rolim Muro MARTINEZ, Sandra Cristina Gonçalves MARTINEZ, Saulo Nardy NADER, Luciana de Oliveira NEVES, Ivan Hideyo OKAMOTO, Rogério Adas Ayres de OLIVEIRA, Fabiano de Melo PEIXOTO, Cristiana Borges PEREIRA, Roberta Arb SABA, Leticia Pereira de Brito SAMPAIO, Lucas Porcello SCHILLING, Marcus Tulius Teixeira SILVA, Emanuelle Roberta SILVA, Jerusa SMID, Cristiane Nascimento SOARES, Manoel SOBREIRA-NETO, Nise Alessandra de Carvalho SOUSA, Leonardo Cruz de SOUZA, Hélio Afonso Ghizoni TEIVE, Vera Cristina TERRA, Matheus VALE, Vitor Mendes Grise VIEIRA, Edmar ZANOTELI, Gilmar PRADO

**Affiliations:** 1 Universidade de São Paulo, Hospital das Clínicas, Faculdade de Medicina, Departamento de Neurologia, São Paulo SP, Brazil. Universidade de São Paulo Universidade de São Paulo Hospital das Clínicas Faculdade de Medicina São Paulo SP Brazil; 2 Hospital Santa Marcelina, Departamento de Neurologia, São Paulo SP, Brazil. Hospital Santa Marcelina Departamento de Neurologia São Paulo SP Brazil; 3 Hospital Sírio-Libanês, Núcleo de Neurociências, São Paulo SP, Brazil; Hospital Sírio-Libanês Hospital Sírio-Libanês Núcleo de Neurociências São Paulo SP Brazil; 4 Hospital Heliópolis, Departamento de Neurologia, São Paulo SP, Brazil. Hospital Heliópolis Departamento de Neurologia São Paulo SP Brazil; 5 Universidade Estadual do Amazonas, Departamento de Neurologia, Manaus AM, Brazil. Universidade do Estado do Amazonas Universidade Estadual do Amazonas Departamento de Neurologia Manaus AM Brazil; 6 Universidade de São Paulo, Faculdade de Medicina de Ribeirão Preto, Ribeirão Preto SP, Brazil. Universidade de São Paulo Universidade de São Paulo Faculdade de Medicina de Ribeirão Preto Ribeirão Preto SP Brazil; 7 Universidade Estadual Paulista “Júlio de Mesquita Filho”, Faculdade de Ciências Médicas e Biológicas de Botucatu, Hospital das Clínicas, Departamento de Neurologia, Psicologia e Psiquiatria, Botucatu SP, Brazil. Universidade Estadual Paulista Júlio de Mesquita Filho Universidade Estadual Paulista “Júlio de Mesquita Filho” Faculdade de Ciências Médicas e Biológicas de Botucatu Hospital das Clínicas Botucatu SP Brazil; 8 Faculdade de Medicina do ABC, Departamento de Neurociências, Santo André SP, Brazil. Faculdade de Medicina do ABC Faculdade de Medicina do ABC Departamento de Neurociências Santo André SP Brazil; 9 Hospital Geral de Goiânia Dr Alberto Rassi, Departamento de Neurologia, Goiânia GO, Brazil. Hospital Geral de Goiânia Dr Alberto Rassi Departamento de Neurologia Goiânia GO Brazil; 10 Santa Casa de Belo Horizonte, Departamento de Neurologia, Belo Horizonte MG, Brazil. Santa Casa de Misericórdia de Belo Horizonte Santa Casa de Belo Horizonte Departamento de Neurologia Belo Horizonte MG Brazil; 11 Universidade Federal de Minas Gerais, Hospital das Clínicas, Departamento de Neurologia, Belo Horizonte MG, Brazil. Universidade Federal de Minas Gerais Universidade Federal de Minas Gerais Hospital das Clínicas Departamento de Neurologia Belo Horizonte MG Brazil; 12 Universidade do Estado do Amazonas, Escola Superior de Ciências da Saúde, Manaus AM, Brazil. Universidade do Estado do Amazonas Universidade do Estado do Amazonas Escola Superior de Ciências da Saúde Manaus AM Brazil; 13 Universidade Federal de Santa Catarina, Santa Catarina RS, Brazil. Universidade Federal de Santa Catarina Universidade Federal de Santa Catarina Santa Catarina RS Brazil; 14 Universidade Federal do Rio de Janeiro, Instituto de Neurologia Deolindo Couto, Departamento de Neurologia, Rio de Janeiro RJ, Brazil. Universidade Federal do Rio de Janeiro Universidade Federal do Rio de Janeiro Instituto de Neurologia Deolindo Couto Departamento de Neurologia Rio de Janeiro RJ Brazil; 15 Universidade Estadual de Campinas, Departamento de Neurologia, Campinas SP, Brazil. Universidade Estadual de Campinas Universidade Estadual de Campinas Departamento de Neurologia Campinas SP Brazil; 16 Hospital das Clínicas, Recife PE, Brazil. Hospital das Clínicas Recife PE Brazil; 17 Universidade Estadual de Campinas, Instituto de Biologia, Genética, Imunologia e Bioagentes, Campinas SP, Brazil. Universidade Estadual de Campinas Universidade Estadual de Campinas Instituto de Biologia, Genética, Imunologia e Bioagentes Campinas SP Brazil; 18 Faculdade de Medicina do ABC, Departamento de Neurologia, Santo André SP, Brazil. Faculdade de Medicina do ABC Faculdade de Medicina do ABC Departamento de Neurologia Santo André SP Brazil; 19 Hospital Israelita Albert Einstein, São Paulo SP, Brazil. Hospital Israelita Albert Einstein Hospital Israelita Albert Einstein São Paulo SP Brazil; 20 Universidade Federal de Ciências da Saúde de Porto Alegre, Departamento de Clínica Médica, Porto Alegre RS, Brazil. Universidade Federal de Ciências da Saúde de Porto Alegre Universidade Federal de Ciências da Saúde de Porto Alegre Departamento de Clínica Médica Porto Alegre RS Brazil; 21 Hospital Moinhos de Vento, Serviço de Neurologia e Neurocirurgia, Porto Alegre RS, Brazil. Hospital Moinhos de Vento Hospital Moinhos de Vento Serviço de Neurologia e Neurocirurgia Porto Alegre RS Brazil; 22 Universidade Anhanguera, Campo Grande MS, Brazil. Universidade Anhanguera Universidade Anhanguera Campo Grande MS Brazil; 23 Hospital Federal dos Servidores do Estado, Departamento de Neurologia, São Paulo SP, Brazil. Hospital Federal dos Servidores do Estado Departamento de Neurologia São Paulo SP Brazil; 24 Universidade Estadual de Campinas, Departamento de Neurologia, Campinas SP, Brazil. Universidade Estadual de Campinas Universidade Estadual de Campinas Departamento de Neurologia Campinas SP Brazil; 25 Hospital da Restauração, Departamento de Neurologia, Recife PE, Brazil. Hospital da Restauração Departamento de Neurologia Recife PE Brazil; 26 Hospital São Carlos, Fortaleza CE, Brazil. Hospital São Carlos Fortaleza CE Brazil; 27 Universidade Federal de São Paulo, Departamento de Neurologia, São Paulo SP, Brazil. Universidade Federal de São Paulo Universidade Federal de São Paulo Departamento de Neurologia São Paulo SP Brazil; 28 Hospital do Servidor Público Estadual, Departamento de Neurologia, São Paulo SP, Brazil. Hospital do Servidor Público Estadual Departamento de Neurologia São Paulo SP Brazil; 29 Pontifícia Universidade Católica do Rio Grande do Sul, São Lucas Hospital, Instituto do Cérebro, Porto Alegre RS, Brazil. Pontifícia Universidade Católica do Rio Grande do Sul Pontifícia Universidade Católica do Rio Grande do Sul São Lucas Hospital Instituto do Cérebro Porto Alegre RS Brazil; 30 Fundação Oswaldo Cruz, Rio de Janeiro RJ, Brazil. Fundação Oswaldo Cruz Fundação Oswaldo Cruz Rio de Janeiro RJ Brazil; 31 Universidade Federal do Ceará, Faculdade de Medicina, Departamento de Medicina Clínica, Fortaleza CE, Brazil. Universidade Federal do Ceará Universidade Federal do Ceará Faculdade de Medicina Departamento de Medicina Clínica Fortaleza CE Brazil; 32 Hospital Universitário Getúlio Vargas, Departamento de Neuromuscular, Manaus AM, Brazil. Hospital Getúlio Vargas Hospital Universitário Getúlio Vargas Departamento de Neuromuscular Manaus AM Brazil; 33 Universidade Federal de Minas Gerais, Faculdade de Medicina, Grupo de Pesquisa em Neurologia Cognitiva e do Comportamento, Belo Horizonte MG, Brazil. Universidade Federal de Minas Gerais Universidade Federal de Minas Gerais Faculdade de Medicina Grupo de Pesquisa em Neurologia Cognitiva e do Comportamento Belo Horizonte MG Brazil; 34 Universidade Federal do Paraná, Hospital de Clínicas, Serviço de Neurologia, Curitiba PR, Brazil. Universidade Federal do Paraná Universidade Federal do Paraná Hospital de Clínicas Serviço de Neurologia Curitiba PR Brazil; 35 Hospital Nossa Senhora das Graças, Epicentro, Curitiba PR, Brazil. Hospital Nossa Senhora das Graças Epicentro Curitiba PR Brazil; 36 Faculdade de Medicina do ABC, Departamento de Neurologia, Santo André SP, Brazil. Faculdade de Medicina do ABC Faculdade de Medicina do ABC Departamento de Neurologia Santo André SP Brazil; 37 Universidade Federal de São Paulo, Escola Paulista de Medicina, Departamento de Neurologia, São Paulo SP, Brazil. Universidade Federal de São Paulo Universidade Federal de São Paulo Escola Paulista de Medicina Departamento de Neurologia São Paulo SP Brazil

**Keywords:** Cannabis, Cannabinoids, Neurology, Cannabidiol, Cannabis, Canabinoides, Neurologia, Canabidiol

## Abstract

Cannabinoids comprehend endocannabinoids, phytocannabinoids, and synthetic cannabinoids, with actions both in the central and peripherical nervous systems. A considerable amount of publications have been made in recent years, although cannabis has been known for over a thousand years. Scientific Departments from the Brazilian Academy of Neurology described evidence for medical use in their areas. Literature is constantly changing, and possible new evidence can emerge in the next days or months. Prescription of these substances must be discussed with patients and their families, with knowledge about adverse events and their efficacy.

## RESUMO

Os canabinoides compreendem os endocanabinoides, fitocanabinoides e os canabinoides sintéticos e desempenham ações no sistema nervoso central e periférico. Uma quantidade enorme de publicações tem sido lançada nos últimos anos, embora a *cannabis* seja conhecida por milênios. Os Departamentos Científicos da Academia Brasileira de Neurologia descreveram as evidências do uso médico em suas áreas. A literatura está em constantes mudanças e possíveis novas evidências podem surgir nos próximos dias ou meses. A prescrição dessas substâncias deve ser discutida com os pacientes e suas famílias, com conhecimento sobre eventos adversos e sua eficácia.

## INTRODUCTION

The term cannabinoids refer to a heterogeneous group of compounds classified into three main groups: endogenous, synthetic, and phytocannabinoids^1^. Phytocannabinoids consist of terpenophenolic substances derived from the *Cannabis sativa* plant. The plant produces more than 100 cannabinoids, including Δ9-tetrahydrocannabinol (THC), the one responsible for its main psychoactive effects, whereas cannabidiol (CBD) is the major non-psychotomimetic compound^1^^,^^2^.

The endocannabinoid system in the central nervous system (CNS) comprises mainly the cannabinoid CB1 and CB2 receptors, their endogenous agonists, the endocannabinoids (EC) anandamide (AEA) and 2-arachydonoilglycerol (2-AG), and the proteins responsible for their uptake, synthesis, and degradation. Cannabinoid receptors are linked to membrane hyperpolarization. As a consequence, the probability of neurotransmitter release from the presynaptic terminal decreases, characterizing the EC as retrograde messengers. These two EC are released by excitatory synaptic activity in response to increased intracellular calcium. They inhibit neurotransmitter liberation from gabaergic and glutamatergic terminals; and act in many mechanisms of inhibitory and excitatory synaptic plasticity^2^^,^^3^^,^^4^.

There are many other systems involved in the EC system, acting as modulators and metabolic processes resulting in an EC-related network, the endocannabinoidome^2^. These models and mechanism actions could explain the CBD potential medical use in many neurological diseases^2^^,^^5^.

There are two synthetic THC approved by the Food and Drug Administration (FDA) and the Medicines and Healthcare products Regulatory Agency (MHRA) of England, nabilone (Cesamet^TM^), used as a post-chemotherapy antiemetic, and dronabinol (Marinol^TM^), for the treatment of anorexia associated with HIV/AIDS and as antiemetic substance.

In 2015, the Brazilian Academy of Neurology had published the first position paper gathering all the evidence on the neurological use of cannabinoids. Some scientific departments have written their statements. Many studies have been published since then, with a significant improvement in knowledge in this field. For the second time, the Brazilian Academy of Neurology is issuing new statements through their departments, including evidence of benefits in neurological diseases^6^.

All scientific departments (SD) (coordinators, vice coordinators, secretaries) were invited to contribute to this positioning paper. Each SD was responsible for each session of this manuscript, assuming their position concerning the use of cannabis-based products.

The Brazilian regulatory National Health Surveillance Agency (*Agência Nacional de Vigilância Sanitária* - ANVISA) has published, in 2019, a resolution that defines the conditions and procedures for manufacturing and importing. This resolution establishes requirements for the commercialization, prescription, dispensing, monitoring, and inspection of Cannabis products for medicinal purposes for human use (RDC 327/2019).

On March 10^th^, 2020, Resolution 327/2019 (ANVISA) was released, which allows the marketing of Cannabis-based products in pharmacies across the country. On April 22^nd^, the first company obtained authorization to produce and distribute the oil, which will only be sold to people with a medical prescription. The rules are based on THC concentration, formulations with a THC concentration higher than 0.2% (type B prescription), or for those with higher than 0.2% THC, only for terminal patients or those who have no therapeutic alternatives (type A prescription, valid for 30 days).

Resolution RDC335, of January 24^th^, 2020, defines the criteria and procedures for the import of cannabis-derived products by individuals, for their use, with a prescription by a legally qualified professional, for health problems.

There is an increasing interest on studies regarding neurological action and therapeutic use of cannabinoids; and an excellent theoretical basis for its use. Many types of research are conducted, and results have been published.

## CANNABIS: THE PLANT AND THE MEDICINE. A HISTORICAL PERSPECTIVE

### Cannabis: origin and spread

The history of hemp (cannabis) and its use dates back to about the 3^rd^ millennium BC in written history, and according to paleobotanical studies, possibly to circa 12 millennia back. The plant has been appreciated for its varied uses, such as fiber, rope, cloth, paper, food, medicine, religion, and recreational^7^^,^^8^.

The origin of this plant is generally placed in Central Asia, and from there, it is believed to have spread, over five millennia, to China, India, Japan, Persia, Arabia, Europe, Africa, and the Americas^8^^,^^9^^,^^10^^,^^11^.

The hemp plant is known by many names, like marijuana, hashish, dagga, bhang, locoweed, grass, *maconha*, *cañamo*, etc. It was first identified and labeled by Carl *Linnaeus* (*Cannabis sativa Linnaeus 1753*). Later, two other distinct species were recognized (*Cannabis indica Lamarck 1785*, and *cannabis ruderalis Janischevsky 1924*). They differ fundamentally in terms of size and content of psychoactive molecules^7^^,^^10^^,^^12^^,^^13^.

### Psychoactive properties discovery

The psychoactive properties of cannabis were known to the Aryans (3,000-600 BC), who introduced the plant to the Scythians, Thracians, and Dacians, whose 'shamans' burned cannabis flowers to induce trance. The Scythians (2,000-1,400 BC) often inhaled the vapors of hemp-seed smoke, both as a ritual and for recreation, as reported by Herodotus (490-425 BC). Through the Aryans, the ancient Assyrians (900 BC) also knew such effects^9^^,^^10^ and used it in some religious ceremonies, they called *qunubu* [*kanab*] (meaning “way to produce smoke”), then converted to Greek as *κάνναβις* (*kánnabis*), a Scythian or Thracian word, and subsequently to Latin as *cannabis*, the origin of the modern word^9^^,^^10^^,^^14^.

### Pharmacological developments

Cannabis’ chemistry is complex and contains a large number of active compounds, many working on the 'EC system,' localized in the central and peripheral nervous system^7^^,^^15^ (Chart 1).

**Chart 1.** Pharmacological developments - chemical composition of cannabis and the endocannabinoid system.

**Cannabis composition.** The plant contains a large number of chemically active compounds, such as cannabinoids, terpenoids, flavonoids, and alkaloids. The most active components, which give its peculiar characteristics, are the ‘cannabinoids.’ Once considered the main active constituent in cannabis, ‘cannabinol,’ was isolated in the 1890s. Later, in the 1930s, ‘cannabidiol’ (CBD) was obtained. Then, in 1964, the primary psychoactive substance Δ-9-THC (l-delta-9-trans-tetrahydrocannabinol), was identified^24^^,^^25^. Subsequently, other cannabinoids were recognized, with some biological effects of their own or by modifying the results of Δ-9-THC. Currently, 538 natural compounds from Cannabis sativa are known, and more than 100 are identified as phytocannabinoids, which can be divided into ten subclasses, among which cannabigerol, cannabichromene, cannabidiol, tetrahydrocannabinol, Δ-9-tetrahydrocannabivarin, cannabicyclol, cannabinol, and other similar compounds. The proportion of these substances in the plant varies according to the species and to where it is grown. Thus, in temperate climates, the plant contains a small proportion of Δ-9-THC (with psychoactive properties) and a relatively high one of CBD (without psychoactive properties), while in hot climates (grown for its psychoactive effects), it contains a high proportion of delta-9-THC and relatively little CBD^6^^,^^7^^,^^9^. **Endocannabinoid system.** The site of action of the cannabinoids is the ‘endocannabinoid system,’ localized in the central and peripheral nervous systems, consisted of endogenous ligands, receptors, and synthesis and degradation enzymes, as proposed by Di Marzo et al. (1994)^15^. Its main receptors, comprising cannabinoid type-1 (CB1) and cannabinoid type-2 (CB2), were identified respectively in 1990 and 1993, and at the same period, the two endogenous CB receptor ligands were also discovered (AEA and 2-AG)^7^^,^^25^.

### History of therapeutic use

Medicinal cannabis (or medical cannabis) concerns the use of *Cannabis sativa* (and of other species) - defined as the unprocessed plant, a part of it, or a plant-derived preparation - for therapeutic purposes^16^^,^^17^.

The medical use of cannabis dates back about 3,000 years BC, in China, prescribed for fatigue, rheumatism, and malaria. Its use as a medicine is extensively reported in the Egyptian Ebers Papyrus dated to about 3,000 years ago, and in Assyrian clay tablets (>650 BC). Its medical use in India probably began around 1,000 years BC, as an analgesic, anticonvulsant, hypnotic, and tranquilizer. Historical and archeological evidence suggests that, in Europe, Scythian invaders brought the plant in 450 BC^10^^,^^13^^,^^16^^,^^18^. It was also well known among the ancient Greeks and Romans, as mentioned by Herodotus (about 400 BC)^7^^,^^13^. Its use in the Americas probably began in South America, reaching Brazil in the XVI century, brought by African slaves, mainly Angolans^13^^,^^18^. The effective introduction of cannabis in Europe occurred in the midst of the XIX century through the works of the French psychiatrist Jacques-Joseph Moreau, who wrote on the psychological effect of its use, and the Irish physician Willian Brooke O’Shaughnessy, who described its use for rheumatism, convulsions, and mainly for muscular spasms of tetanus and rabies. The medical use of the drug spread from Britain and France, reaching all Europe and then North America^13^^,^^18^.

Following a rapid rise of cannabis usage in 1900s medicine, it began to decline due to controversies over legal, ethical, and societal implications, therapeutic indications based on limited clinical data, as well as anti-cannabis laws, which practically abolished any modern efforts to investigate possible therapeutic applications of the medicine^18^^,^^19^. However, in the past two decades, an increasing interest was seen in the therapeutic potential of cannabis derivatives for neurological disorders, with a strong stimulus for research in several areas, such as epilepsy (Lennox-Gastaut and Dravet syndromes), multiple sclerosis (MS) symptoms, neuropathic pain, movement disorders (*e.g*., Parkinson disease - PD), dementias (*e.g*., Alzheimer disease - AD), among other manifestations^6^^,^^19^^,^^20^.

Regarding Brazil, one must remember the pioneering studies of Carlini’s group, with cannabis and tetrahydrocannabinols^21^, followed by reviews of Zuardi et al.^13^ and, more recently, studies from the same group, coordinated by Chagas et al., emphasizing the use of CBD in patients with neurological diseases (PD)^22^^,^^23^.

Despite persistent controversies, the use of cannabis for medicinal purposes represents the revival of a plant with long historical significance reemerging in present-day health care. The properties of cannabis foreshadow transformations of neurological treatment into a new reality of effective interventional and even preventative care^10^^,^^13^.

## CANNABINOIDS AND THE BLOOD-BRAIN-BARRIER

Potential therapeutic actions of the cannabinoids delta-9-THC and CBD are based on their activity as an anti-inflammatory, anti-seizure, as well as analgesic and antiemetic. Results from laboratory and human studies suggest that it could be a promising experimental animal model of epilepsy and produce antipsychotic effects in experimental novel agents for CNS diseases, including schizophrenia and epilepsy. Due to THC and CBD lipophilicity and their neurological actions, they are natural candidates as new medicinal approaches to treat CNS diseases. However, their penetrability and disposition in the brain are different, and these patterns are related to their role in the blood-brain-barrier (BBB). Several findings indicate that CBD can modify the deleterious effects on BBB caused by inflammatory cytokines and may play a pivotal role in ameliorating BBB dysfunction consequent to ischemia and hyperglycemia. Cannabinoids can positively influence the brain’s immune response, playing a role in the prevention of BBB damage. In this regard, it has been hypothesized that the activation of the EC system could play a key role in preventing interactions between immune and endothelial cells and in neuroprotection through the maintenance of tight junctions. These findings suggest that CBD could be part of a new strategic approach useful to treat inflammatory diseases of the CNS^24^^,^^25^^,^^26^^,^^27^^,^^28^.

Cannabinoids may also interfere in BBB pump-efflux regulation, playing an essential role in drug resistance in the clinical management of neurological or psychiatric diseases such as epilepsy and schizophrenia^27^^,^^28^^,^^29^.

In addition to their use as therapeutic agents in epilepsy, pain, and movement disorders, cannabinoids effects upon BBB may justify their use in neurodegenerative diseases. Cannabinoids’ positive impact on cognitive function could be considered through the aspect of protection of BBB cerebrovascular structure and function, indicating that they may purchase substantial benefits through the protection of BBB integrity.

Emerging evidence suggests beta-amyloid (Aβ) deposition in the AD brain is the result of impaired clearance, due in part to diminished Aβ transport across the BBB. The modulation of the cannabinoid system may reduce Aβ brain levels and improve cognitive behavior in AD animal models. Bachmeier and coworkers investigated the role of the cannabinoid system in the clearance of Aβ across the BBB using *in vitro* and *in vivo* models of BBB clearance. They examined Aβ transit across the BBB in the presence of cannabinoid receptor agonists and inhibitors and determined the expression levels of the Aβ transport protein, lipoprotein receptor-related protein1 (LRP1) in the brain and plasma of mice following cannabinoid treatment. Cannabinoid receptor agonism or inhibition of endocannabinoid-degrading enzymes significantly enhanced Aβ clearance across the BBB (2-fold).

Moreover, cannabinoid receptor inhibition negated the stimulatory influence of cannabinoid treatment on Aβ BBB clearance. Additionally, LRP1 levels in the brain and plasma were elevated following cannabinoid treatment (1.5-fold), providing a rationale for the observed increase in Aβ transit from the brain to the periphery. These findings provide an insight into the mechanism by which cannabinoid system modulation has been shown to reduce Aβ brain burden and abrogate AD pathophysiology and cognitive decline^30^^,^^31^.

Still, within the scope of cognitive impairment and BBB integrity, it is known that type-2 diabetes (T2D) increases the risk of dementia by 5-fold, and evidence suggests that the heightened inflammation and oxidative stress in T2D may lead to disruption of the BBB, which precedes premature cognitive decline. Compromised integrity of the BBB in T2D is both an early and critical event preceding cognitive decline and potentially dementia, so targeting the BBB may be a novel therapeutic approach for diabetes-associated dementia. Brook et al., in a recent review, point out the role of BBB dysfunction in T2D associated dementia and consider the potential therapeutic use of cannabinoids as a protectant of cerebrovascular BBB preventing neurocognitive impairment^26^.

### Cannabinoids in epilepsy (children and adults)

The first records of the use of marijuana for medical purposes date back to 2737 BC in China^32^. However, the EC system was only discovered in 1992^33^. The global interest in the use of medical marijuana in epilepsy grew exponentially in the 21^st^ century after Charlotte Figi’s story gained notoriety in the United States of America^34^, and Anny Fischer’s, in Brazil. Since then, several articles produced without strict scientific structure have been published, many of them just roughly drafted, and until 2014, Cochrane and American Academy of Neurology reviews did not show scientific evidence that would substantiate the use of marijuana for epilepsy^35^^,^^36^. Still, CBD has been investigated and found useful in the treatment of patients with epilepsy for several decades, particularly in the pediatric age group. In recent years, there has been a growing interest in the use of CBD as an adjunctive treatment in patients with refractory epilepsy^37^.

A controlled series of patients being treated with Epidiolex - drug comprised of CBD, a compound derived from marijuana and indicated for epilepsy - was published. At this time, Devinsky et al.^38^ showed the results for 214 patients treated with highly pure (99%) CBD in 10 epilepsy centers in the United States from January 2014 to January 2015, including children and young adults, with severe forms of epilepsy. This study showed a median reduction of 36.5% in the frequency of seizures per month.

In 2017 Devinsky et al.^39^ conducted a double-blind, placebo-controlled, randomized trial to evaluate the efficacy of Epidiolex for seizures in 120 children and adult patients with Dravet syndrome. In another double-blind, placebo-controlled study, Thiele et al. assessed the effectiveness of Epidiolex for atonic seizures in 225 patients with Lennox-Gastaut syndrome. This study showed that patients who experienced a reduction of at least 50% in the drop seizure frequency were mostly in the CBD group, 43%, compared to 27% in the placebo group^40^. These two randomized, controlled studies which evaluated the efficacy of pharmaceutical-grade CBD in children with Dravet and Lennox-Gastaut syndromes showed similar efficacy to other antiepileptic medications^39^^,^^40^. CBD was approved for use in the treatment of patients with epilepsy with the diagnosis of Lennox-Gastaut Syndrome and Dravet Syndrome by the FDA in 2018 and by the European Medicines Agency (EMA) in 2019. The approval was based on the previous commented studies in which CBD, as an addiction drug, was shown to be superior to placebo in reducing epileptic seizures^38^^,^^39^^,^^40^. In these studies, the median percentage reduction from baseline in seizure frequency was 44%, data statistically significant when compared to the placebo group. Based on these studies, the FDA approved the first commercial presentation of highly purified CBD (Epidiolex®; GW Pharmaceutical, Cambridge, UK).

CBD has also been shown to be effective in the treatment of patients with focal epilepsy associated with other clinical conditions, such as Tuberous Sclerosis Complex^40^. Still, in these patients, CBD has an off-label indication. The recommended dose of CBD for refractory epilepsy treatment was 10 to 25 mg/kg/day (about 200 to 300 mg/day) ^37^.

More recently, de Carvalho Reis et al. systematically examined the efficacy and adverse events profile of CBD and medicinal cannabis. They observed a statistically meaningful effect of CBD compared to placebo. Furthermore, CBD proved more effective than a placebo, regardless of the etiology of the epileptic syndromes and dosage^41^.

Concerning the efficacy of CBD for epilepsy, a study that assessed its use for patients with tuberous sclerosis should also be emphasized. After three months of treatment, patients who took CBD experienced a mean reduction of 48.8% in seizure frequency and, after 12 months, a decrease of 50% or more in 50% of the patients. It was also observed that among patients who took clobazam (CLB) (12/18) concomitantly, the response rate was 58.3%, compared to 33.3%^10^ in patients who did not take it^42^.

In all mentioned studies, adverse events were frequent, occurring in almost 90% of patients^40^. The most common adverse events observed were somnolence, decreased appetite, diarrhea, behavioral changes, skin rash, fatigue, convulsive episodes, status epilepticus, lethargy, gait disorder, sedation, as well as changes in the dosages of concomitant antiepileptic medications^38^^,^^39^^,^^40^^,^^41^^,^^42^^,^^43^. These events were described as mild or moderate, with a small percentage of patients discontinuing treatment^38^. Severe adverse events were reported in up to 30% of patients, including one case of sudden unexpected death in epilepsy (SUDEP), although regarded as unrelated to the study drug^41^. Adverse events in CBD using were more common under short-term than under long-term treatment^41^.

Animal studies demonstrate adverse effects related to the male reproductive system, with reduced spermatogenesis, changes in embryological and fetal development, reducing peripheral organ weight and neurotoxicity^43^, and its use in a gestational age patient should be considered with thrift.

CBD has complex and variable pharmacokinetics, with a low oral bioavailability, which increases up to four times when ingested with a high-fat diet^44^. Metabolism is hepatic, and CBD did not significantly modify plasma levels of most antiepileptic drugs (slightly increases the serum level of phenobarbital and phenytoin and reduces the serum level of ethosuximide)^45^. The exception is the association of CBD and CLB, which is particularly effective, with a bidirectional interaction, in which both have an inhibitory action on metabolism, with CBD inhibiting the metabolism of CLB and its primary metabolite, and CLB inhibiting metabolism of CBD and its metabolite, 7-hydroxy-CBD^46^. This association leads to a serum level increase of both drugs, enhancing their therapeutic effect, but with a potential increase in side effects such as drowsiness, sedation, respiratory hypersecretion, and infections^43^^,^^47^. The significant consequence of this interaction is somnolence, which can be addressed by reducing the dose. Concerning other medications, the interactions seem to be less evident, albeit not completely known.

There may also be an increase in liver enzymes when CBD is associated with sodium valproate^48^. Some studies have shown an increase of ≥3 times the upper limit of normal in the serum levels of alanine or aspartate aminotransferase for approximately 15% of patients taking CBD, which proved to be the main reason for the treatment to be discontinued. There is a potential risk of hepatoxicity, increased by the concomitant use of valproate. In all the cases, the laboratory abnormalities could be reversed by reducing the dose of one concomitant antiepileptic medication, mainly valproate or CLB, or after the reduction or discontinuation of CBD^49^^,^^50^^,^^51^.

An increase in the levels of zonisamide, eslicarbazepine acetate, topiramate, and rufinamide could be observed with the concomitant use of CBD^50^^,^^51^.

The interaction of CBD with antiepileptic medications seems common in patients with epilepsy, especially with clobazam and valproate^49^. These two medications may significantly influence the levels of efficacy and safety of CBD and must be carefully considered in daily medical practice.

Currently available CBD presentations are marketed as supplements and therefore are not subject to regulatory regulations for medicines. Some presentations, in addition to containing high concentrations of THC, do not have the correct level of CBD. Thus, the Mayo Clinic suggests that a checklist should be made to assess whether the chosen presentation is reliable, considering: production quality control (manual of good manufacturing practices, and organic certificate with European, Australian or Canadian standards), international certification by the National Science Foundation); assess whether the company has an independent program to report adverse effects; the product must have organic certification and be tested, guaranteeing a THC concentration >0.3%, without pesticides and heavy metals^52^.

Despite all new antiepileptic drugs that have been developed in recent years, approximately 30% of patients with epilepsy continue with their seizures uncontrolled. In this context, current scientific data allow us to infer that CBD has a potential role in the treatment of these patients. However, drug interactions, safety profile, and efficacy are not yet proven^6^. In this way, we must discuss with patients/family the indications of off-label CBD use, considering potential risks and benefits, and choose formulations that have a higher content of CBD with THC levels below 0.3% (Chart 2).

**Chart 2.** Cannabidiol and epilepsy.

In summary: 1. The efficacy of CBD in the treatment of patients with epilepsy is similar to that of other antiepileptic medications; 2. CBD is more effective in convulsive episodes, especially in certain childhood epileptic syndromes such as Dravet and Lennox-Gastaut; 3. CBD causes adverse events, what may limit its use; 4. CBD may be an alternative in refractory epilepsy; 5. The use of CBD must be limited to the well-known pharmaceutical drugs. 6. Consider potential risks and benefits, discuss with patients/family the indication of off-label CBD use.

### Cannabinoids in multiple sclerosis

The use of cannabis in MS started as a complementary symptomatic treatment, especially for those symptoms not entirely controlled by standard therapies. Several patients admit recurrent use of products derived from cannabis (PDC) to relieve symptoms such as spasticity, pain, insomnia, anxiety, ataxia, and tremor. In a cohort at the University of British Columbia in Vancouver, Canada, a city where recreational marijuana use is allowed, a study found that around 30% of patients interviewed used some cannabis-derived product to treat pain, insomnia, moodiness, or spasticity without their doctor’s knowledge. Some side effects reported were forgetfulness and lack of attention. Interestingly, 35% of these PDC users had never tried traditional symptomatic medications, and 56% had previously tried only one symptomatic standard therapy. The reasons for this search for alternative treatments are still unclear and, therefore, this behavior should be studied^53^.

Cannabinoid type 1 (CB1) and type 2 (CB2) receptors are expressed in the central and peripheral nervous system and in the immune system. CB1 is more expressed in the CNS in areas associated with pain control, as well as the cerebellum, hippocampus, peripheral nerves, dorsal root ganglion, and neuromuscular junction. CB1 stimulation decreases neurotransmitter release, affecting nociceptive pathways, memory, psychic activity, and motor control. CB2 receptors are expressed in cells of the immune system as macrophages, neutrophils, and lymphocytes, which explains some of the anti-inflammatory activity of cannabinoids, which can also stimulate other receptors such as opioid and serotonergic receptors. THCs have high affinities for the CB1 receptor, which explains their actions on psychic activity, including a change in mood and consciousness. In contrast, CBD has little affinity for CB1 and CB2 receptors and may assume an antagonistic role by competing with them in the presence of THC, decreasing their potency. The main effect of CBD is on non-cannabinoid receptors, including ion channels^2^.

There are no consistent studies for the therapeutic indication of cannabis in the form of cigarettes in any of the symptoms of MS. A recent meta-analysis indicated a slight efficacy of the treatment for spasticity, pain, and urinary retention in patients with MS (pwMS). But in most of them, the primary endpoint of these studies was based on subjective self-assessment scales. Objective scales, such as Ashworth’s applied by neurologists, did not show improvement in spasticity. Another issue is that there are different PDCs with different formulations being tested, for example, products with only synthetic or natural THC or even mixtures of THC and CBD in different ratios such as 1:1 or 2:1 rate, which prevented a real comparison of their effectiveness and side-effects^54^^,^^55^^,^^56^^,^^57^^,^^58^^,^^59^.

There are class I, II, and III studies of compounds extracted from cannabis to treat some different symptoms, such as spasticity and pain. More than 85% of pwMS can suffer from some type of spasticity during their lifetime, which can contribute to their disability, especially in the more advanced stages of the disease. A preparation combining THC and CBD, in the ratio of 1:1, exclusively for oral use and used in the maximum dose of up to 12 puffs per day, nabiximol (Sativex^TM^) showed an improvement of more than 20% in the spasticity parameters after four weeks of use compared to placebo. He then received FDA approval for the treatment of severe and refractory spasticity in MS^57^^,^^59^.

In neuropathic or central pain, it can affect around 70% of patients, in the form of headache (43%), neuropathic pain in the upper or lower limbs (26%), low back pain (20%), painful spasms (15%), and trigeminal neuralgia (3.8%). The studies were carried out in short periods, with variable efficacy. Nabiximols showed improvement in pain when compared to placebo in patients with MS. Another study demonstrated effectiveness in controlling chronic neuropathic pain in the association of nabilone with gabapentin. Oral cannabis extracts have shown conflicting results, and although it is not possible to conclude their effectiveness definitively, these data suggest that this may be a therapeutic option in patients who have not responded to conventional treatments^60^^,^^61^.

In the treatment of tremors, the use of nabiximols or oral preparations of THC, CBD or THC/CDB, proved to be ineffective, and there is currently no indication for its use to relieve these symptoms. For urinary symptoms, nabiximols showed a likely improvement in reducing the frequency, but with no effect on urinary incontinence^59^.

Retrospective studies indicate some benefit of using CBD for anxiety and insomnia after one month of treatment. These results suggest that this therapeutic option can be considered in pwMS, however, controlled and long-term studies need to be carried out to prove its real efficacy and safety^58^^,^^59^.

Some precautions must be taken regarding the indication of PDC in MS, as their side effects can be aggravated due to the peculiarities of the disease. Symptoms such as cognitive impairment, fatigue, and mood changes, which can vary from depression to suicidal ideation, must be evaluated before indicating these substances in MS. The main side effects of cannabis use are mainly related to THC and high dosage used, which include vertigo, drowsiness, and nausea. In the long run, it can affect cognition and balance. The most severe effects are induction of psychosis and schizophrenia in at-risk individuals, heart disease (myocardial infarction, hypertension, heart failure, and stroke), and cannabinoid hyperemesis syndrome, which can be enhanced if associated with smoking^53^^,^^54^^,^^56^^,^^59^.

Mainly, regarding cognitive functions, patients are vulnerable to a time-dependent decline, control of disease activity, and type of MS (remitting-recurrent or progressive forms), affecting their quality of life and work capacity. Studies comparing patients who use PDC (inhaled or ingested) with those who do not use it demonstrated a significant worsening in information processing speed, working memory, and cognitive functions. Those who use PDC are twice as likely to have changes in the neuropsychological assessments of those who do not. Proving these findings, a recent study assessing patients who extensively used cannabis and who had previously altered neuropsychological tests, demonstrated significant improvement 28 days after ceasing its use in all cognitive domains. In addition, it was shown by functional brain magnetic resonance imaging (MRI) associated with the symbol digit modality test (SDMT), increased blood oxygen level-dependent activation (BOLD) in 4 regions of the neural network involved with SDMT performance after the cessation of cannabis use. In the phase 3 study of the effectiveness of nabiximol to treat spasticity, the cognitive functions evaluated by the PASAT test did not change with treatment during the study. However, more complete neuropsychological tests are needed to assess the presence of this side effect^62^^,^^63^.

### Cannabinoids in movement disorders

Recently, there has been a growing interest in the medicinal use of cannabinoid derivatives in treating PD and other movement disorders.

Some studies have been published to seek a definitive answer on the use of cannabinoid derivatives, especially CBD, in patients with abnormal movements, with a greater interest in patients with PD due to its high prevalence.

One of the first studies carried out in PD patients treated with CBD for four weeks demonstrated a decrease in psychotic symptoms without worsening motor function or inducing adverse effects^64^. Another study showed that although CBD does not improve the motor function of patients with PD or their overall symptom score, treatment for six weeks improves the quality of life of these patients, suggesting that this effect may be related to anxiolytic, antidepressant, and CBD antipsychotic drugs^63^^,^^65^. In 2015, Kluger et al. published a review of the preclinical and clinical studies that existed until then on the therapeutic potential of cannabinoids in various movement disorders. The conclusion was that there is not enough data to indicate certain benefits from the use of these substances in patients with involuntary movements, such as tics, dystonia, and blushing^66^. A similar conclusion was found for patients with PD because, although observational and uncontrolled studies suggest some positive response in motor symptoms (tremor and bradykinesia), these results have not been reproduced in controlled studies. For dystonia, two controlled studies with a small number of patients showed no benefit in the abnormal movements of patients treated with CBD compared to the control group^67^^,^^68^. A recent review showed that regarding motor symptoms, only one study, with a small sample of patients, demonstrated the benefit of CBD use for treat L-Dopa induced dyskinesia^69^.

In conclusion, despite the widespread by the lay media of the possible benefits of cannabinoids in movement disorders, especially PD, there are reports of some improvement in non-motor symptoms such as psychosis, sleep disorders, and pain, as well as improvement in scales that assess quality of life, there are no scientific data to support this indication. Most of the studies are uncontrolled, with a small number of patients, short follow-up, and without data on cognition and long-term evolution. The few existing controlled studies have shown no effect on PD motor symptoms, nor in patients with chorea or dystonia.

### Cannabinoids in chronic pain

Chronic pain affects 28% of the general population, and it is the leading cause of years lived with disability from all diseases worldwide^70^. There are several chronic pain syndromes, grouped according to the primary pathophysiological mechanism related to their occurrence. They are classified as:

- Nociceptive/inflammatory pains.
- Neuropathic pain (central or peripheral).
- Nociplastic pain (primary headaches, fibromyalgia, and nonspecific low back pain)^71^.

In one study, chronic pain was the leading cause of seeking a physician’s prescription for medical marijuana^72^. However, evidence for the routine use of cannabinoids in chronic pain is still limited^73^. In some pain syndromes, such as peripheral neuropathic pain, the evidence leans against its effectiveness, especially considering a large number of first-, second-, and third-line treatments available, which were already approved and known to be useful for the treatment of this pain syndrome^74^.

Nevertheless, the use of cannabinoids may be proposed for the treatment of some specific cases in which there is no well-established evidence-based treatment, such as spinal cord injury-related pain, central post-stroke pain syndromes, neuropathic pain related to the use of chemotherapy or due to traumatic peripheral nerve. Additionally, the prescription must be based on a clear rationale, as an adjuvant treatment, and within an individualized plan treatment.

As with opioid prescriptions, patients should be frequently monitored to detect possible adverse behavioral, mood, appetite, or systemic events (*i.e*., bronchitis, risk of an accident while driving). Unlike opioids^75^, there are still no straightforward ways to identify patients at higher risk of developing cannabinoid-related abuse or addiction disorder. Therefore, care must be taken in instances of dysfunctional (nociplastic) pain syndromes (*i.e*., fibromyalgia, primary headaches), personal history of abuse of licit or illicit drugs, or personal/family history of severe psychiatric illness. In these situations, substance misuse is more frequent, regardless of their pharmacological classes, and thus special attention should be paid.

Frequently, patients with chronic pain may seek cannabinoid prescription for relief of symptoms that are not directly related to their pain, but rather to improve sleep, anxiety, concentration, mood, well-being, or muscle relaxation. The identification of this primary objective is essential to analyze whether cannabinoids would be the best treatment available for the demand and, thus, guide patients to the most appropriate form of pharmacological or non-pharmacological treatment. This “targeting of the bothersome symptoms” is also essential to monitor the real effects of cannabinoids, should they be initiated.

As with any psychotropic agent, when prescribing cannabinoids, careful history taking should be performed to accurately identify the patient’s demands, needs, and expectations regarding the treatment and use of this class of medication. Currently, the monthly cost of most cannabinoids legally approved for clinical use is high, and this factor needs to be taken into account when prescribing a drug for use in the medium and long term.

### Cannabinoids in muscular diseases

The role of the endocannabinoid system (ECS) in muscle physiology was initially identified in animal models as responsible for the functions related to energy expenditure and glucose uptake^76^^,^^77^. In addition to this role related to muscle energy dynamics, Iannotti and collaborators later demonstrated, through studies using gene silencing techniques associated with pharmacological tools, that the ECS has, mainly via stimulation of CB1 receptors by endogenous or exogenous cannabinoids, effects on the proliferation of myoblasts^78^^,^^79^^,^^80^.

In this sense, it is possible to infer that the ECS possibly influences the pathophysiology of several myopathies, especially in muscular dystrophies. Studies with an animal model of Duchenne muscular dystrophy (DMD) treated with phytocannabinoid agents - CBD, cannabivarin (CBDV), and tetrahydrocannabivarin (THCV) - showed a sustained improvement in the motor performance of treated animals when compared to controls^81^. Among the possible mechanisms involved are the anti-inflammatory effect, the autophagy recovery, and also an increase in the myoblast’s differentiation^81^. Considering their impact in human satellite cells, myotubes generation in both healthy and DMD muscle samples was seen not only with CBD and CBDV but also with THCV^82^. Another cannabinoid derivate, tetrahydrocannabinol, was investigated in animal models regarding its effects in reducing acute muscle pain. Both local and systemic administration had an antinociceptive effect^83^.

Evidence from animal models has shown that CB2 receptors play a role in the inflammatory muscle response during the muscle repair course. Its activation attenuates the inflammatory response and favors the anti-fibrotic/pro-fibrotic balance in the muscle repair process, whereas its blockade results in the opposite effects^84^. Additional CB2 receptor functions may be seen in regenerated myotubes from muscle ischemia-reperfusion models where they seem to play a protective effect by relieving oxidative stress and accelerating early myogenesis. The levels of CB2 receptor protein were also higher during the differentiation of C2C12 myoblasts, an immortalized mouse myoblast cell line, suggesting the participation of the CB2 receptor in the muscle regeneration process^85^.

This evidence regarding the immune response modulation may also find a direct effect on the pathophysiology of inflammatory myopathies, for example. *In vitro* evidence of the action of a CB2 receptor agonist agent on peripheral blood mononuclear cells of patients with dermatomyositis (DM) demonstrate its ability to reduce the secretion of IL-31 (interleukin related to the innate and adaptive immune response in the skin)^86^. This same CB2 agonist agent proved to be tolerable and safe for patients with DM in phase II studies^87^^,^^88^ and, at the moment, a phase III study, for this same patient profile, is in progress^89^.

Considering the available *in vivo* evidence supporting the use of cannabinoids for myopathies, apart from the phase II studies mentioned for patients with DM, the scientific background of their possible benefit in humans is restricted only to a few reports/case series^90^^,^^91^^,^^92^. That said, it is paramount to state that, in the present moment, there is no sufficient evidence to recommend a systematic cannabinoid prescription for myopathic patients.

### Cannabinoids in neurological rehabilitation

The indication and use of cannabinoids in a neurological rehabilitation environment is hugely restricted, with no established level of evidence for this use, except for patients with spasticity such as paraplegias or MS. Spasticity is a deficit that impacts on worsening functional capacity, resulting in problems in activities of daily living. Chronically, spasticity can lead to muscle pain, spasms or stiffness, reducing mobility, and to contracture leading to bone and joint deformities. Evidence in the literature is moderate about the impact of cannabinoids on spasticity due to MS or paraplegia, as well as adverse events such as dizziness, drowsiness, and nausea. A larger number of randomized clinical trials are needed to evaluate cannabinoids for spasticity and chronic pain in this patient population, as well as for other indications^93^.

Future studies on the use of CBD derivatives, such as Sativex, are being carried out to assist in neurological rehabilitation with a focus on improving spasticity, trunk control, and patient gait, associated with rehabilitation training with robotic assistance^94^.

In the environment of patients undergoing neurorehabilitation who progress to palliative care or end-of-life care, cannabinoids can be used exceptionally, despite the more significant experience of use still being in oncological diseases. The purpose of use in this scenario would be to control pain. However, there is still limited evidence of the role of this drug in patients with neurodegenerative diseases, also indicated for the improvement of symptoms other than pain, such as sleep problems, fatigue, anxiety, and depression, nausea, and vomiting. Side effects of using cannabinoids are drowsiness, dizziness, dry mouth, anxiety, euphoria, paranoia, toxic psychosis, tachycardia, orthostatic, hypotension, slow reaction time, headache, blurred vision, cognitive impairment, and depression, with around 20% of patients discontinuing treatment due to side effects^95^.

### Cannabinoids in dementia

There is evidence suggesting that cannabinoids may modulate core pathophysiological mechanisms of AD, such as amyloidosis and tau-related neurodegeneration. Data from both animal and *in vitro* studies indicate that cannabinoids may reduce the hyperphosphorylation of Tau protein^96^. Moreover, it seems that cannabinoids may also reduce the production of beta-amyloid peptide^96^. Interestingly, cannabinoids may regulate microglial activation, leading to reduced neuroinflammation and oxidative stress^96^^,^^97^, which have been recently recognized as key neurobiological processes related to the pathophysiology of AD. Although interesting, it should be pointed out that these results come from experimental studies with animal and *in vitro* models; these findings have not been demonstrated in clinical practice.

Few studies have investigated the possible benefit of cannabinoids in the management of patients with dementia. Observational studies suggest that cannabinoids may be useful in the symptomatic control of behavioral changes in patients with AD or other dementias^97^^,^^98^. However, there are no randomized, multicenter, double-blind studies with a large number of patients supporting the use of these drugs in clinical practice. There is no evidence that cannabinoids slow the clinical progression of AD. Therefore, eventual benefits are essentially symptomatic and not curative.

In summary, although some results are suggesting that cannabinoids may be of scientific interest in neurodegenerative diseases, there is a lack of evidence to support their clinical use in patients with dementia. Therefore, their use is not recommended for treating patients with AD and related disorders. Further studies are warranted to establish the clinical value of cannabinoids in dementia practice.

### Cannabinoids and sleep disorders

The use of cannabinoids for sleep disorders has been tested in a few clinical trials (CT). Most of the CT are about studies in patients with insomnia, but some investigated the use of CBD in obstructive sleep apnea and, rarely, in other disorders as parasomnias. A summary of these findings is stated below:

### Insomnia

In a meta-analysis involving 19 CT, a total of 3,231 patients with insomnia associated with various comorbidities (chronic pain, fibromyalgia, MS, etc.) had been evaluated, using different types of cannabinoids: Nabiximol (13 studies); Sativex (THC/CBD) (2 studies); THC (inhaled cannabis) (2 studies); dronabinol/nabilone (2 studies)^99^. However, only two of them had a low risk of bias. The meta-analysis found 8 CT that demonstrated improvement in sleep quality with the use of cannabinoids from 2 to 15 weeks compared to placebo (weighted mean difference [WWD] -0.58, 95%CI -0.87 to -0, 29). The benefit was seen mainly with nabiximol^99^^,^^100^.

In a crossover CT with 32 patients with insomnia associated with fibromyalgia, the use of nabilone 0.5 mg daily compared to 10 mg amitriptyline improved insomnia symptoms (mean difference from baseline, -3.25, 95%CI -5.26 to -1.24) and the perception of restorative sleep (mean difference from baseline, 0.48, 95%CI 0.01-0.95) in the 2 weeks that followed^100^.

There is little evidence that the use of cannabinoids, such as Nabiximol and Nabilone, improves short-term sleep quality in patients with insomnia associated with chronic pain, fibromyalgia, and MS. However, no evidence supports its use for insomnia disorder.

### Obstructive sleep apnea

There are two controlled placebo CT with a total of 95 patients^101^^,^^102^. One of them involved 22 patients with obstructive sleep apnea (OSA) and showed a greater benefit of using dronabinol (maximum dose of 10 mg/day) *versus* placebo in reducing the rate of sleep apnea/hypopnea (AHI) (mean line difference base -19.64, p=0.02) with three weeks of follow-up. The other study involved 73 adult patients with moderate or severe OSA who received a placebo (n=25), 2.5 mg dronabinol (n=21), or 10 mg dronabinol (n=27) daily, 1 hour before sleep during six weeks. In comparison with placebo, dronabinol reduced dose-dependent AHI by 10.7±4.4 (p=0.02) and 12.9±4.3 (p=0.003) events/hour, with doses of 2, 5, and 10 mg/day, respectively^102^.

Although promising preliminary results with dronabinol were showed, there is still no reliable evidence that favors the use of cannabinoids for OSA treatment^103^.

### Parasomnias

Two CT using nabilone *versus* placebo demonstrated a reduction of more than 70% in the frequency of nightmares associated with post-traumatic stress syndrome^104^^,^^105^.

An open study with four patients with REM behavior disorder (RBD) associated with PD, the use of cannabidiol (75-300 mg) led to a complete reduction of symptoms in three out of the four patients^106^.

There is still no clear evidence to demonstrate the benefits of using cannabinoids to treat parasomnias. More CT with a larger number of patients and longer follow-up are needed.

### Cannabinoids in traumatic brain injury

The neuroprotective antioxidant effects of cannabinoids are particularly relevant in their ability to counteract “glutamate excitotoxicity,” which leads to neuronal demise after traumatic brain injury (TBI). Anecdotally, cannabis, particularly chemovars combining THC and CBD, has been beneficial in the treatment of chronic traumatic encephalopathy (CTE) symptoms: headache, nausea, insomnia, dizziness, agitation, substance abuse, and psychotic symptoms. CTE, previously known as dementia pugilistica, or “punchdrunk syndrome” has garnered a great deal of attention due to its apparent frequency among long-term American football players but including victims of repetitive head injury from causes as diverse as other contact sports, warfare and even “heading” in soccer.

Neuroprotective benefits of phytocannabinoids, particularly CBD, further outlined below, provide support for trials of these agents in post-traumatic syndrome and CTE prevention^107^.

However, as in other neurological diseases, more research is needed, especially controlled studies with long-term follow-up, to support these results.

### Cannabinoids in vestibular disorders

The use of cannabinoids for vestibular disorders has not been tested in human clinical trials (CT). Most of the information about the ECS and the vestibular system is based on studies in mice^108^^,^^109^. The pharmacological actions of cannabinoids in the context of nausea and vomiting are limited. More research is needed to understand their effectiveness in the treatment of nausea and vomiting.^110^^,^^111^^,^^112^ There are not CT about cannabinoids and dizziness treatment^113^. On the other hand, dizziness and vertigo are commonly reported adverse side-effects in CT of medical cannabinoid ^113^^,^^114^^,^^115^ The are case reports that smoking cannabis can suppress pendular nystagmus in patients with MS^115^^,^^116^. Anecdotal and empirical use of CBD for some refractory vestibular disorders was reported by some of the authors of this review, but the results are not consistent.

There is a lack of evidence about the medical use of CBD for vestibular disorders and nausea; Dizziness is a possible side effect of the medicinal use of CBD.

## CANNABINOIDS IN NEUROINFECTION

### HIV

Several factors impact the quality of life of HIV patients, including vomits, anorexia, and pain. Anorexia in these patients can be caused by stomatitis, intermittent or chronic diarrhea, and opportunistic infections, such as cytomegalovirus, microsporidia, and cryptosporidium. Increased appetite and vomit control are fundamental for a better prognosis. Dronabinol, a synthetic form of THC, directly acts in the vomiting and appetite control centers in the brain, thereby increasing appetite and preventing vomiting^117^. Although it has been used for the treatment of anorexia associated to weight loss in patients with HIV/AIDS, indicators like increasing appetite, reducing nausea, and improving functional status were mostly assessed in single studies, and associations failed to reach statistical significance^118^.

Neuropathic pain due to HIV-associated sensory neuropathy is the most common peripheral nerve disorder complicating HIV infection. It is directly associated with a decrease in daily functioning in HIV-infected individuals. Two clinical trials assessed the impact of smoked cannabis on neuropathic pain in HIV; both studies found that smoked cannabis was well tolerated and effectively relieved chronic neuropathic pain from HIV-associated sensory neuropathy (achieving at least or greater than 30% pain relief) ^119^^,^^120^.

There is low-quality evidence that cannabinoids studies improve weight gain in HIV patients and moderate-quality evidence that it improves neuropathic pain in these patients.

### Cannabinoids in headache

There are no clinical studies that support the therapeutic use of cannabinoids in any of the main primary headaches, such as migraine, tension-type headache, and cluster headache. In migraine, studies in an animal model have shown contradictory results, whereas in cluster headaches, there are only isolated reports of efficacy^121^^,^^122^^,^^123^^,^^124^^,^^125^^,^^126^. Even though some painful neuropathies of the cephalic segment, such as trigeminal neuralgia, burning mouth syndrome, and persistent idiopathic facial pain seem to respond to the use of cannabinoids, their use is not recommended by the guidelines or consensus regarding the treatment of headache, due to the lack of conclusive data.

The prescription of medical cannabinoids must be discussed with each patient and family. Neurologists have to know adverse effects and possible pharmacological interactions among prescribed medications. There are many cannabinoids compounds with few known information, which could be promising in many neurological diseases. We need to clarify misunderstood conceptions about the medical use of cannabidiol and derivative synthetic cannabinoids. We have to research cannabinoids’ actions in neurological disorders, with randomized, blind, and controlled trials to confirm their beneficial effects, remembering that many usually prescribed substances for some conditions are used off-label.

### Safety in using Cannabis-based treatment

Use of cannabis-based products can be associated with several adverse events and these must be considered before prescribing them as a medicines. The adverse events may be related to the route of administration or to its content, but also to user’s behavior, drug to drug interactions, family history, and other medical conditions. One of the challenges in prescribing a cannabis-based treatment, besides the lack of evidence for some alleged indication, is the fact that some of the preparations of cannabis-based medicine are faced as herbal medicine and considered harmless.

A basic knowledge from the prescriber is expected concerning the associated risks in these therapies. Different onset of action, duration and self-titration possibilities are observed depending on the administration route, faster to slower in smoked, vaporized, inhaled, oil, oil-capsule, and edible respectively, as quick as 5 min to few hours to initiate the effect, and 2 to 12 hours of duration (Table 1). Because of its slow start of action there is an increased risk of higher dosage in edibles presentation, despite some patient ideas of a safer use experience with edibles^127^.

**Table 1.** Cannabinoids route of administration and characteristics.

Route of administration	Onset (min)	Duration (h)	Amenable to self-titration
Smoked	5	2‒4	++++
Vaporized	5	2‒4	++++
Oral (oil, capsule, edible)	30‒60	8‒12	+
Oromucosal	15‒40	2‒4	++

Cannabinoids should be avoided as much as possible in patients below 25 years-old, in those with family history of psychotic symptoms, in patients that describe a bad experience in previous exposition to marijuana, or who are heavy drinkers, heavy tobacco smoking, with poor cardiac conditions, hypotension, and not to be used in pregnant, in those who intend to get pregnant or breastfeeding women^128^.

Respiratory complications in inhaled or vaporized forms as rhinitis, chronic bronchitis, and pulmonary hemorrhage and coagulopathy in synthetic presentations of inhaled cannabinoids are described^129^^,^^130^. Local injury in oromucosal after prolonged use of THC/CBD oromucosal spray is referred by some MS patients. This usually is transitory and prompted recovered after a brief interruption^131^.

The THC/CBD content is not uniform throughout all the presentations, leading to a higher risk of a lower or a higher dosage comparing with the desired one. Cannabinoids interaction with liver metabolized drugs, using several enzymes of the P450 CYP system, is well known^132^. Amongst antiseizure medications there is evidence showing an increase in levels of topiramate, rufinamide, zonisamide, eslicarbazepine and N-desmethylclobazam, this last one with increased sedation, in epilepsy patients^51^. Concomitant use of anticoagulants like warfarin carries an increase in bleeding risk. Other associations to be avoided are St John wort (*Hypericum perforatum*), rifampin, ketoconazole, and some antiviral drugs^51^.

Another concern regarding cannabis-based medicine is the driving skills. It is well known that THC impairs the ability to drive, and the time lapse between THC use and safety drive is considered between six to eight hours after consumption^133^^,^^134^. That may not be the case for pure CBD content medicines, but few studies addressed that at this moment. A 2018 literature review evaluating the driving skills specifically in MS patients having THC/CBD mucosal spray for spasticity treatment did not find any evidence of increase in motor vehicle-accident in those patients, with reference to improvement in motor and cognitive abilities related to driving^134^.

The Lower-Risk Cannabis Use Guidelines (LRCUG) published in 2017 summarizes behaviors that can decrease or increase the associated risk of cannabis use and is a good line to follow to offer a safer experience to cannabis treated patient^128^.

Finally, while we wait for good evidence publications, in favor or against cannabis medicine, knowing the mechanisms of action, the pharmacology, the restrictions and contraindications may be more helpful to our patients than been excited to any new biased indication.
